# Retraction Note: Pumpkin seeds (*Cucurbita pepo* subsp. *ovifera*) decoction promotes *Trichinella spiralis* expulsion during intestinal phase via “Weep and Sweep” mechanism

**DOI:** 10.1038/s41598-025-15100-x

**Published:** 2025-08-13

**Authors:** Aml S. Saleh, Samah A. El-Newary, Walaa A. Mohamed, Abdelbaset M. Elgamal, Mona A. Farah

**Affiliations:** 1 Zoology Department, Faculty of Women for Arts, Science and Education, Ain Shams University, Cairo, Egypt; 2 Medicinal and Aromatic Plants Research Department, Pharmaceutical and Drug Industries Research Institute, National Research Centre, 33 El Bohouth St. (Former EL Tahrir St.), Dokki, P.O. 12622, Giza, Egypt; 3 Pharmaceutical and Drug Industries Research Division, Department of Chemistry of Microbial and Natural Products, National Research Centre, Giza, Egypt

Retraction of: *Scientific Reports* 10.1038/s41598-024-51616-4, published online 18 January 2024

The Editors have retracted this Article. After publication, concerns were raised regarding the data presented in Figure 4. Specifically, six panels in Figure 4 appear to overlap with the panels presented in Figure 1 in [1]. These Panels represent tissues taken from animals subject to different experimental conditions. The Authors were unable to provide the original raw data for the full set of panels. The Editors therefore no longer have confidence in the results and conclusions reported.

Aml S. Saleh did not explicitly state if they agree or disagree with the retraction. The remaining Authors did not reply to correspondence from the Editors about this retraction.
